# CircFBXW4 Suppresses Colorectal Cancer Progression by Regulating the MiR‐338‐5p/SLC5A7 Axis

**DOI:** 10.1002/advs.202300129

**Published:** 2024-03-10

**Authors:** Wei Song, Jincheng Fu, Jing Wu, Jun Ren, Rensheng Xiang, Can Kong, Tao Fu

**Affiliations:** ^1^ Department of Gastrointestinal Surgery II Renmin Hospital of Wuhan University Wuhan 430060 P. R. China; ^2^ Department of General Surgery Qingdao Municipal Hospital Qingdao 266071 P. R. China

**Keywords:** circFBXW4, colorectal cancer, miR‐338‐5p, SLC5A7

## Abstract

Dysregulated circular RNAs (circRNAs) contribute to tumourigenesis and cancer progression. However, the expression patterns and biological functions of circRNAs in colorectal cancer (CRC) remain elusive. Here, RNA sequencing and bioinformatics analyses are applied to screen for aberrantly expressed circRNAs. The expression of circFBXW4 in CRC tissues and cell lines is determined by quantitative real‐time PCR. A series of in vitro and in vivo biological function assays are implemented to assess the functions of circFBXW4. The regulatory mechanisms linking circFBXW4, miR‐338‐5p, and SLC5A7 are explored by western blotting, dual luciferase reporter assays, and RNA pull‐down assays. CircFBXW4 is dramatically downregulated in CRC tissues and cell lines. circFBXW4 downregulation is clearly correlated with malignant features and patient overall survival in CRC. Functionally, ectopic expression of circFBXW4 strikingly impairs the proliferation, migration, and invasion capacities of CRC cells in vitro and in vivo, whereas circFBXW4 knockdown has the opposite effects. Mechanistically, circFBXW4 competitively binds to miR‐338‐5p and prevents it from interacting with and repressing its target SLC5A7, thus suppressing the progression of CRC. This study reveals the specific critical role of circFBXW4 in inhibiting CRC progression via the miR‐338‐5p/SLC5A7 axis and provides an additional target for eradicating CRC.

## Graphical abstract

A novel circular RNA, circFBXW4, is characterized here which is downregulated in colorectal cancer (CRC). Mechanistically, circFBXW4 work as a miR‐338‐5p sponge to abolish its inhibitory effect on SLC5A7, thereby suppressing CRC tumorigenesis, growth, and metastasis. Collectively, targeting circFBXW4 is a promising strategy for CRC treatment. This work expands the knowledge of molecular mechanism underlying CRC and might offer a novel target for precision medicine of CRC.

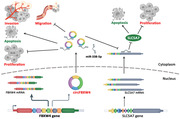

## 1 Introduction

Colorectal cancer (CRC) is the second most common cause of cancer deaths and accounts for ≈10% of all cancers.^[^ ^1^ ^]^ With changes in risk factors, early screening, resection of precancerous polyps during colonoscopy, and implementation of comprehensive treatment regimens based on surgery, the incidence and mortality of CRC have gradually stabilized or even decreased.^[^ ^2^, ^3^ ^]^ When CRC is diagnosed at an early stage, surgery is the primary treatment modality. However, ≈20% of CRC patients have metastatic disease at diagnosis, and up to 50% of patients with initially localized disease eventually develop metastases.^[^ ^4^, ^5^ ^]^ The overall prognosis for patients with metastatic CRC is poor, and the 5‐year survival rate is less than 20%.^[^ ^5^ ^]^ For these patients, systemic therapy is the main treatment option. Patients initially respond well to these treatments but then often develop resistance.^[^ ^4^, ^6^, ^7^ ^]^ In addition, 35% to 40% of patients with KRAS and NRAS gene mutations do not respond to targeted therapy.^[^ ^5^ ^]^ CRC is a heterogeneous disease caused by a series of progressive histological changes, each of which is accompanied by specific genetic alterations.^[^ ^8^ ^]^ There is an urgent need to elucidate the molecular mechanisms underlying CRC initiation and progression and identify new biomarkers and targets for the diagnosis and treatment of this disease.

Circular RNAs (circRNAs) are a novel class of endogenous noncoding RNAs (ncRNAs) generated from precursor messenger RNAs (mRNAs) mainly via direct backsplicing or exon skipping.^[^ ^9^, ^10^ ^]^ CircRNAs were initially considered meaningless “byproducts” of the splicing process or gene rearrangements.^[^ ^11^ ^]^ This view changed with the advent of RNA sequencing (RNA‐seq), and it was found that circRNAs could accumulate to levels exceeding those of mRNAs and be widely expressed in a tissue‐specific or development‐specific manner.^[^ ^12^, ^13^, ^14^, ^15^ ^]^ CircRNAs are not only highly conserved, stable, abundant, and expression specific but are also widely present in various bodily fluids. These properties indicate the powerful biomarker potential of circRNAs.^[^ ^16^, ^17^ ^]^ Emerging evidence suggests that most well‐characterized circRNAs to date play a functional role in gene expression regulation; mechanistically, they act by binding to RNA‐binding proteins (RBPs), functioning as microRNA (miRNA) sponges, or constituting templates for the translation of specific peptides or proteins.^[^ ^18^, ^19^, ^20^ ^]^ Dysregulated expression of circRNAs has been observed in several cancer types and exerts important effects on tumourigenesis, progression, and metastasis.^[^ ^21^, ^22^, ^23^ ^]^ However, only a few circRNAs with known functions and mechanisms in CRC have been well recognized, and additional circRNAs with unknown functions are worth further exploration. Therefore, it is necessary to search for circRNAs specifically expressed in CRC and to further address their mechanisms of action and pathophysiological roles. The knowledge gained will help to reveal the pathogenic mechanisms of CRC and drive the discovery and development of potential biomarkers/therapeutic targets.

One circRNA with an unknown function in CRC – has_circ_0 008362, also called circFBXW4 – is a circRNA located on chromosome 10. Mouse circFBXW4 (mm9_circ_000338) was first reported and characterized by Chen et al.,^[^ ^24^ ^]^ who found that the expression of mm9_circ_000338 was significantly downregulated in the fibrogenesis stage but restored in the hepatic fibrosis stage in the mouse liver. Lower levels of circFBXW4 were also confirmed in hepatic fibrosis patients than in healthy controls. Functional experiments revealed that enforced expression of mm9_circ_000338 inhibited hepatic stellate cell activation and proliferation, induced apoptosis, attenuated fibrogenic injury in the mouse liver and showed anti‐inflammatory effects. This study also showed that mm9_circ_000338 may act as a suppressor of hepatic stellate cell activation and hepatic fibrosis by directly targeting the miR‐18b‐3p/FBXW7 axis. Geng et al.^[^ ^25^ ^]^ found that circFBXW4 was associated with tumor grade in patients with glioma. However, neither characterization of circFBXW4 nor related functional experiments were performed in this study. Indeed, a characterization of circFBXW4 was first reported in a study on recurrent spontaneous abortion.^[^ ^26^ ^]^ The study also explored the physiological functions of circFBXW4 in trophoblast cells and found that it could inhibit the proliferation and invasion of a trophoblast cell line by sponging miR‐324‐3p. However, the functions of circFBXW4 in the progression of CRC are largely unknown.

Here, we show for the first time that circFBXW4 functions as a novel tumor suppressor in CRC. We verified that circFBXW4 is downregulated in CRC cells and samples and that its expression is correlated with tumor differentiation, stage, and patient prognosis. We further demonstrated that circFBXW4 inhibits tumor growth and metastasis both in vitro and in vivo. Mechanistically, circFBXW4 acts as a sponge of miR‐338‐5p to upregulate SLC5A7 and inhibit tumor progression. Our findings indicate that the circFBXW4/miR‐338‐5p/SLC5A7 axis is a potential therapeutic target for CRC.

## 2 Results

### 2.1 CircRNA Expression Profiles in CRC Tissues

**Figure 1:**
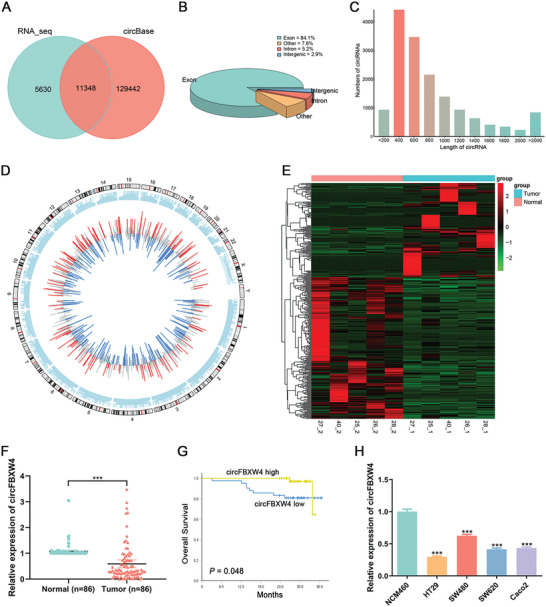
Expression profiles of circRNAs in CRC tissues and cell lines. A) Overlap of circRNAs between our RNA‐seq data and circBase. B) Categories of the detected circRNAs in terms of genomic origin. C) Numbers of detected circRNAs of different lengths. D) Distribution of the detected circRNAs on human chromosomes and expression of the circRNAs. The length of a line represents the relative magnitude of the fold change, and blue and red indicate downregulated and upregulated circRNAs, respectively. E) Heatmap and volcano plot visualizing the DEcircRNAs in CRC tissues relative to the matched adjacent normal tissues. Green and red indicate low and high expression, respectively. F) The relative expression of circFBXW4 in CRC tissues and matched adjacent normal tissues was determined by qRT‒PCR (n = 86). G) Kaplan‒Meier curve showing the OS of 86 patients with CRC. The patients were dichotomized by the median expression level of circFBXW4. H) The relative expression of circFBXW4 in colon cell lines was determined by qRT‒PCR. The data are shown as the means ± SDs; ***P < 0.001.

To obtain relatively comprehensive circRNA expression profiles in CRC, we performed RNA‐seq analysis on randomly selected tumour tissues and adjacent normal tissues from 5 patients with stage II or III CRC who underwent radical resection. As shown in **Figure** **1**A, 16978 circRNAs were detected, among which 5630 were newly discovered and 11348 have been indexed in the circBase database. In terms of genomic origin, most of the detected circRNAs were derived from exons (84.1%) (Figure 1B). Moreover, the length of the circRNAs exhibited an unequal distribution, with most of the circRNAs having a length of less than 1000 nucleotides (Figure 1C). The expression of the circRNAs and their distribution on human chromosomes are shown in a Circos plot (Figure 1D).

Next, we identified the differentially expressed circRNAs (DEcircRNAs) using the edgeR R package. A total of 356 DEcircRNAs were identified (fold change ≥ 1.5 and *P* < 0.05), among which 142 were upregulated and 214 were downregulated (Figure 1E). Then, 6 candidate circRNAs with a fold change of greater than 2, a relatively high expression level, good intragroup consistency, large intergroup differentiation, and exonic origin were identified by a screen. The expression levels of these candidate circRNAs were further verified in 8 pairs of CRC and paracancerous tissues. Quantitative real‐time PCR (qRT‒PCR) analysis demonstrated that hsa_circ_0 008362 expression in CRC tissues was apparently downregulated compared with that in normal tissues (Figure S1, Supporting Information). Hence, hsa_circ_0 008362 (termed circFBXW4) was selected for further experiments.

To further validate whether circFBXW4 expression is downregulated in CRC tissues, qRT‒PCR analysis was performed in a large number of samples. The qRT‒PCR results suggested that circFBXW4 expression in CRC tissues was evidently downregulated (*P* < 0.001, Figure 1F). Low circFBXW4 expression in tumors was associated with a decreased overall survival (OS) time (*P* = 0.048, Figure 1G). We measured the absolute expression of circFBXW4 in normal colon epithelial cell line NCM460 and colon cancer cell lines by absolution quantification (Figure S2A and B, Supporting Information). Subsequently, we detected the relative expression levels of circFBXW4 in these cell lines by the 2^−ΔΔCT^ method, and the lower levels of circFBXW4 in SW480, SW620, HT29, and Caco2 cells relative to NCM460 cells were confirmed (Figure 1H). Among the CRC cell lines, SW480 showed the highest expression of circFBXW4. Therefore, the SW480 cell line was selected for subsequent cell function experiments. In addition, SW480 cells were derived from the primary lesion of a rectal adenocarcinoma patient, while SW620 cells were derived from a metastatic lymph node of the same patient. Therefore, the SW620 cell line was also selected for subsequent study herein.

### 2.2 Characterization and Clinical Significance of circFBXW4

**Figure 2:**
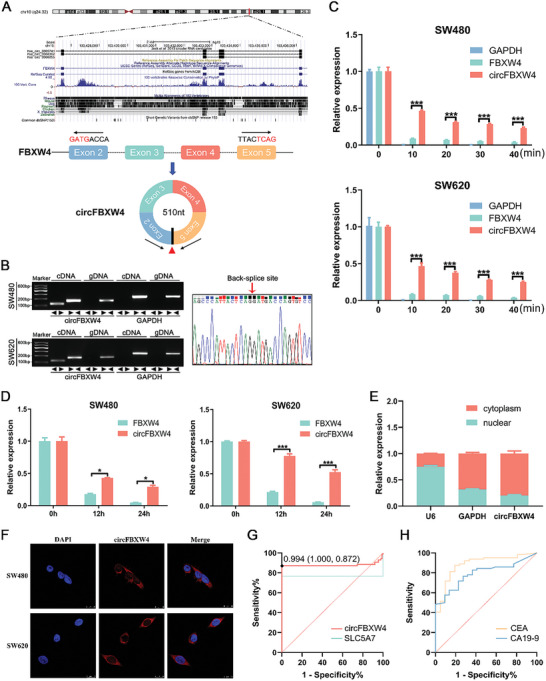
Characterization and clinical significance of circFBXW4. A) Schematic illustration of the genomic location of circFBXW4 and its generation by backsplicing. B) CircFBXW4 was detected in SW480 and SW620 cells by qRT‒PCR with divergent and convergent primers and validated by agarose gel electrophoresis. The head‐to‐tail splicing of circFBXW4 was confirmed by Sanger sequencing. C) The expression of circFBXW4 and linear FBXW4 mRNA in SW480 and SW620 cells treated with RNase R for different durations was analyzed by qRT‒PCR. D) The expression of circFBXW4 and linear FBXW4 mRNA in SW480 and SW620 cells was measured by qRT‒PCR after treatment with actinomycin D for 12 h and 24 h. E) The expression of circFBXW4 in the cytoplasm and nucleus was measured by qRT‒PCR. F) The FISH assay results showed that circFBXW4 was localized predominantly in the cytoplasm in CRC cell lines. Nuclei were stained blue with DAPI, and cytoplasmic circFBXW4 was stained red. G) ROC curves of circFBXW4 and SLC5A7 for distinguishing between CRC tissues and non‐CRC tissues. H) ROC curves of CEA and CA19‐9 for distinguishing between CRC patients and healthy donors. *P < 0.05, ***P < 0.001.

CircFBXW4 is a closed circRNA with a length of 510 nt that is produced by head‐to‐tail splicing of exons 2–5 of the FBXW4 gene (**Figure** **2**A). To validate the backsplicing sequences, we designed divergent and convergent primers for the amplification of circFBXW4 and FBXW4 mRNA, respectively. Using cDNA and gDNA from SW480 and SW620 cells as templates, we found that circFBXW4 was amplified by divergent primers from cDNA but not from gDNA. However, products were amplified by the convergent primers from both the cDNA and gDNA templates. The presence of the head‐to‐tail junction in circFBXW4 was confirmed by Sanger sequencing (Figure 2B). Because they lack free 3′ and 5′ ends, circRNAs are resistant to degradation by RNase R. Thus, the expression of circFBXW4 and the corresponding FBXW4 mRNA was evaluated after treatment with RNase R for different times. The qRT‒PCR results demonstrated that the abundance of FBXW4 was apparently reduced, while circFBXW4 exhibited resistance to digestion by RNase R (Figure 2C). Furthermore, actinomycin D was employed to validate the stability of circFBXW4. CircFBXW4 was more stable than linear FBXW4 mRNA (Figure 2D). Next, the results of qRT‒PCR analysis of the separated nuclear and cytoplasmic fractions and fluorescence in situ hybridization (FISH) confirmed that circFBXW4 was present mainly in the cytoplasm (Figure 2E,F).

To explore the diagnostic role of circFBXW4, a receiver operating characteristic (ROC) curve was drawn based on circFBXW4 expression in CRC tissues. The estimated optimal diagnostic threshold was 0.994, which yielded a sensitivity of 0.872 and a specificity of 1. The area under the ROC curve (AUC) was 0.879 (95% CI: 0.810‐0.943), which was much higher than that for SLC5A7 (AUC of 0.767) (Figure 2G), indicating that circFBXW4 expression can effectively distinguish between CRC tissues and non‐CRC tissues. To further evaluate the diagnostic performance of plasma or plasma exosomal circFBXW4 among those CRC patients. We collected plasma samples from CRC patients and cancer‐free control individuals and detected the circFBXW4 level in plasma. The results showed that circFBXW4 levels in plasma were very low or even undetectable. Therefore, circFBXW4 levels in plasma may not be a diagnostic biomarker for CRC. CEA and CA19‐9 are two widely used clinical biomarkers for CRC diagnosis that were significantly upregulated in patient plasma but had a similarly or even lower AUC (CA19‐9: AUC = 0.885; CEA: AUC = 0.793; Figure 2H) compared with tissue circFBXW4. Serum CEA, CA19‐9, and tissue circFBXW4 complement each other, and the combination of the two may be more comprehensive diagnosis of CRC.

We performed statistical analyses to explore the relationships between circFBXW4 expression and clinicopathological parameters (sex, age, tumor site, differentiation status, histological type, TNM stage, CEA level, microvascular invasion status and perineural invasion status) of CRC patients. As shown in **Table** **1**, circFBXW4 expression was negatively associated with tumor differentiation status (*P* = 0.038) and TNM stage (*P* = 0.018) but was not significantly correlated with sex, age, tumor site, histological type, CEA level, microvascular invasion status or perineural invasion status.

**Table 1:** Correlations between the circFBXW4 expression level and clinicopathological parameters of CRC patients.

Characteristic	CircFBXW4 expression level		*P* value
	Low (N [%])	High (N [%])	
Sex			0.053
Male	20 (47.6)	30 (68.2)	
Female	22 (52.4)	14 (31.8)	
Age			0.941
≤60	14 (33.3)	15 (34.1)	
> 60	28 (66.7)	29 (65.9)	
Tumour site 1			0.540
Colon	25 (59.5)	29 (65.9)	
Rectum	17 (40.5)	15 (34.1)	
Tumour site 2			0.115
Left	27 (64.3)	35 (79.5)	
Right	15 (35.7)	9 (20.5)	
Differentiation status			0.038
Well	2 (4.8)	7 (15.9)	
Moderate	29 (69.0)	34 (77.3)	
Poor	3 (7.1)	0	
Unknown	8 (19.0)	3 (6.8)	
Histological type			0.646
Adenocarcinoma	38 (90.5)	41 (93.2)	
Mucinous adenocarcinoma	4 (9.5)	3 (6.8)	
CEA level			0.264
≤5	31 (73.8)	38 (86.4)	
> 5	10 (23.8)	6 (13.6)	
Unknown	1 (2.4)	0	
TNM stage			0.018
I‐II	17 (40.5)	29 (65.9)	
III‐IV	25 (59.5)	15 (34.1)	
Microvascular invasion status			0.959
Yes	12 (28.6)	12 (27.3)	
No	26 (61.9)	27 (61.4)	
Unknown	4 (9.5)	5 (11.4)	
Perineural invasion status			0.943
Yes	9 (21.4)	10 (22.7)	
No	29 (69.0)	29 (65.9)	
Unknown	4 (9.5)	5 (11.4)	

### 2.3 CircFBXW4 Suppresses CRC Progression In Vitro

**Figure 3:**
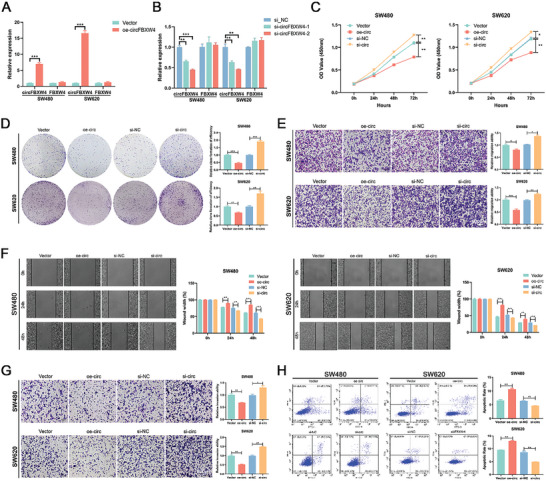
CircFBXW4 suppresses the proliferation, migration, and invasion and promotes the apoptosis of CRC cells in vitro. A) qRT‒PCR analysis of circFBXW4 and FBXW4 mRNA in SW480 and SW620 cells transfected with the circFBXW4‐OE plasmid. B) qRT‒PCR analysis of circFBXW4 and FBXW4 mRNA in SW480 and SW620 cells treated with siRNAs. C) CCK‐8 assays were performed to determine the proliferation ability of CRC cells transfected with the overexpression plasmid or siRNAs. D) Colony formation assays were performed to determine the proliferation ability of CRC cells transfected with the overexpression plasmid or siRNAs. E) Transwell migration assays were performed to determine the migration ability of CRC cells transfected with the overexpression plasmid or siRNAs. F) Wound healing assays were performed to determine the migration ability of CRC cells transfected with the overexpression plasmid or siRNAs. G) Transwell invasion assays were performed to determine the invasion ability of CRC cells transfected with the overexpression plasmid or siRNAs. H) Flow cytometric analysis was performed to determine the effect of circFBXW4 expression on CRC cell apoptosis by Annexin V‐FITC/PI double staining. *P < 0.05, **P < 0.01, ***P < 0.001.

**Figure 4:**
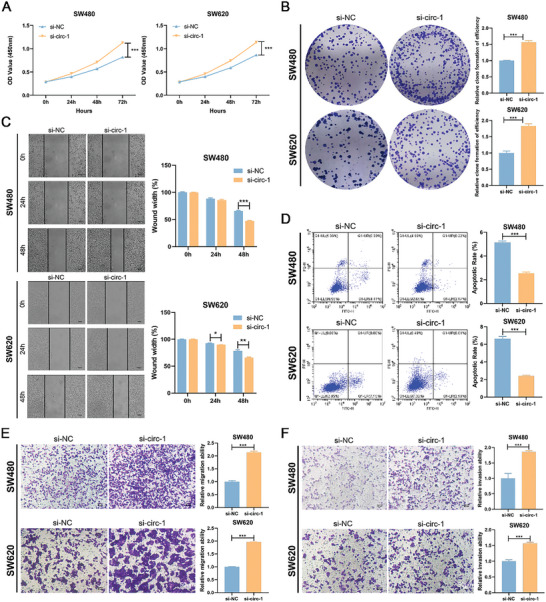
The role of si‐circFBXW4‐1 on cell viability, migratory, invasion, and apoptosis of CRC cells. A) CCK‐8 assays were performed to determine the proliferation ability of CRC cells transfected with the si‐circFBXW4‐1 and si‐NC. B) Colony formation assays were performed to determine the proliferation ability of CRC cells transfected with the si‐circFBXW4‐1 and si‐NC. C) Wound healing assays were performed to determine the migration ability of CRC cells transfected with the si‐circFBXW4‐1 and si‐NC. D) Flow cytometric analysis was performed to determine the effect of circFBXW4 expression on CRC cell apoptosis by Annexin V‐FITC/PI double staining. E) Transwell migration assays were performed to determine the migration ability of CRC cells transfected with the si‐circFBXW4‐1 and si‐NC. F) Transwell invasion assays were performed to determine the invasion ability of CRC cells transfected with the si‐circFBXW4‐1 and si‐NC. *P < 0.05, **P < 0.01, ***P < 0.001.

To evaluate the role of circFBXW4 in CRC cell behaviors, we constructed an overexpression vector specific for circFBXW4 and confirmed by qRT‒PCR analysis that circFBXW4 expression in CRC cells was markedly upregulated, while the expression of linear FBXW4 was not significantly changed (**Figure** **3**A). Subsequently, two small interfering RNAs (siRNAs) for circFBXW4 were synthesized, and the interference efficiency of these constructs was evaluated by qRT‐PCR. The results showed that both siRNAs could silence circFBXW4 in CRC cells without altering the expression of linear FBXW4, and si‐circFBXW4–2 had the highest inhibitory efficiency (Figure 3B). Thus, si‐circFBXW4–2 was chosen for the following experiment. We first checked the effect of circFBXW4 on cell viability. Overexpression of circFBXW4 markedly reduced the viability of SW480 and SW620 cells, while knockdown of circFBXW4 exerted the opposite effects (Figure 3C D). To determine the effect of circFBXW4 on cancer metastasis, Transwell and wound healing assays were performed. The migratory and invasive capabilities of CRC cells were dramatically attenuated by upregulation of circFBXW4 but markedly enhanced by downregulation of circFBXW4 (Figure 3E–G). To determine the role of circFBXW4 in apoptosis, we performed an Annexin V‐FITC/PI apoptosis assay. CircFBXW4 overexpression caused an increase in the percentage of apoptotic cells, while circFBXW4 silencing inhibited apoptosis in CRC cells (Figure 3H). Furthermore, the role of si‐circFBXW4‐1 on cell viability, migratory, invasion, and apoptosis of CRC cells were determined, and similar results were observed (**Figure** **4**). Taken together, these results indicate that circFBXW4 suppresses cell behaviors related to CRC progression in vitro.

### 2.4 CircFBXW4 Functions as a Sponge for miR‐338‐5p in CRC

**Figure 5:**
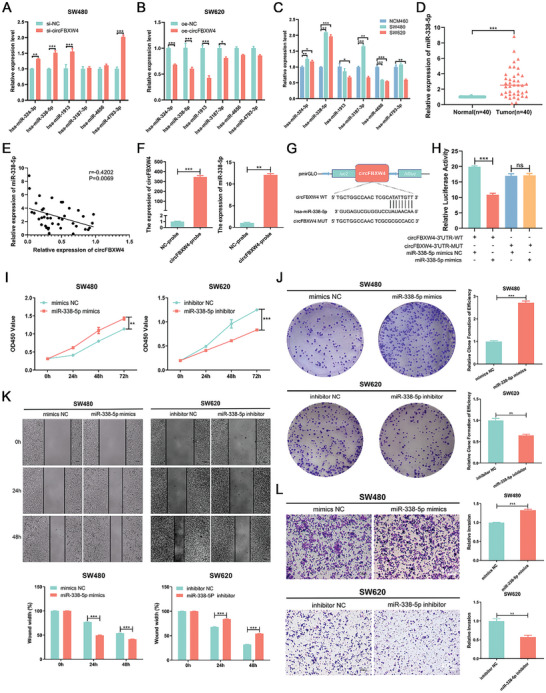
CircFBXW4 functions as a sponge for miR‐338‐5p in CRC. A) The relative expression levels of the candidate miRNAs in SW480 cells transfected with siRNAs were measured by qRT‒PCR. B) The relative expression levels of candidate miRNAs in SW620 cells transfected with the overexpression plasmid were measured by qRT‒PCR. C) The relative expression levels of the candidate miRNAs in NCM460 cells and two CRC cell lines were measured by qRT‒PCR. D) The relative expression of miR‐338‐5p in CRC tissues and matched adjacent normal tissues was determined by qRT‒PCR (n = 40). E) Pearson correlation analysis between the expression levels of circFBXW4 and miR‐338‐5p in our own patient cohort (n = 40). F) The interaction between circFBXW4 and miR‐338‐5p was confirmed by an RNA pull‐down assay. CircFBXW4 and miR‐338‐5p were significantly enriched in the circFBXW4‐Biotin probe group compared with the NC‐Biotin control probe group. G) Schematic illustration of the circFBXW4‐WT and circFBXW4‐MUT luciferase reporter vectors. H) Relative luciferase activity was measured in 293T cells after cotransfection with the circFBXW4‐WT or circFBXW4‐MUT plasmid and the miR‐338‐5p mimic or NC. I) CCK‐8 assays were performed to determine the proliferation ability of CRC cells transfected with the miR‐338‐5p mimic or inhibitor. J) Colony formation assays were performed to determine the proliferation ability of CRC cells transfected with the miR‐338‐5p mimic or inhibitor. K) Wound healing assays were performed to determine the migration ability of CRC cells transfected with the miR‐338‐5p mimic or inhibitor. L) Transwell invasion assays were performed to determine the invasion ability of CRC cells transfected with the miR‐338‐5p mimic or inhibitor. The data are shown as the means ± SDs; *P < 0.05, **P < 0.01, ***P < 0.001.

Given that circFBXW4 is derived from exons of FBXW4 and is predominantly localized in the cytoplasm, we deduced that circFBXW4 may serve as a miRNA sponge. Therefore, we investigated whether circFBXW4 inhibits the progression of CRC by sponging miRNAs. We screened for the possible target miRNAs of circFBXW4 with the TargetScan database, and 6 candidate miRNAs (miR‐324‐3p, miR‐338‐5p, miR‐1913, miR‐3187‐3p, miR‐4656, and miR‐4793‐3p) were comprehensively selected for subsequent validation. After circFBXW4 knockdown, the expression levels of miR‐324‐3p, miR‐338‐5p, miR‐1913, and miR‐4793‐3p were evidently elevated in SW480 cells (**Figure** **5**A), while overexpression of circFBXW4 in SW620 cells significantly reduced the expression levels of these miRNAs (Figure 5B). We further measured the expression of the 6 miRNAs in normal colonic epithelial cells and CRC cells. qRT‒PCR analysis indicated that the expression levels of miR‐324‐3p and miR‐338‐5p were markedly elevated in SW480 and SW620 cells compared to NCM460 cells (Figure 5C). Taken together, these findings suggest that miR‐324‐3p and miR‐338‐5p may be the target miRNAs of circFBXW4. Thus, we selected miR‐338‐5p, whose expression was most significantly affected by the expression of circFBXW4, for subsequent studies. The expression level of miR‐338‐5p was measured by qRT–PCR in 40 paired CRC and adjacent normal tissues, and the results suggested that miR‐338‐5p expression in CRC tissues was evidently upregulated (*P* < 0.001, Figure 5D). Pearson correlation analysis revealed a negative correlation between circFBXW4 expression and miR‐338‐5p expression in CRC tissues (*r* = −0.420, *P* = 0.007, Figure 5E).

To further confirm whether circFBXW4 can directly bind to miR‐338‐5p, we designed a biotin‐labelled circFBXW4 probe and performed an RNA pull‐down assay. As expected, relative to the control oligo probe, the circFBXW4 probe specifically captured circFBXW4 and coprecipitated miR‐338‐5p (Figure 5F). To validate the binding of circFBXW4 and miR‐338‐5p, we conducted a dual luciferase reporter assay. The binding sites in circFBXW4 and miR‐338‐5p were predicted based on the Circular RNA Interactome database, and circFBXW4 wild‐type (circFBXW4‐WT) and mutant (circFBXW4‐MUT) vectors were constructed (Figure 5G). After transfection of the miR‐338‐5p mimic, a dramatic reduction in luciferase activity was observed in cells transfected with the WT reporter but not in those transfected with the mutant reporter (Figure 5H), suggesting that circFBXW4 can directly bind to miR‐338‐5p and negatively regulate its expression.

### 2.5 MiR‐338‐5p Promotes Malignant Behaviors of CRC Cells In Vitro

On the basis of the interaction of circFBXW4 and miR‐338‐5p, we further determined the functions of miR‐338‐5p in CRC cells. Several studies have revealed that elevated miR‐338‐5p expression is positively related to advanced TNM stage and worse prognosis in CRC patients.^[^ ^27^ ^]^ In this study, the results of the CCK‐8 and colony formation assays indicated that increased expression of miR‐338‐5p evidently enhanced the proliferation capacity of SW480 cells, whereas transfection of the miR‐338‐5p inhibitor attenuated the proliferation capacity of SW620 cells (Figure 5I and J). Next, we observed the changes in the migration of CRC cells after transfection of the miR‐338‐5p mimic and inhibitor by a wound healing assay. Evaluation of the cells 24 and 48 h after generation of the wound, the degree of wound healing in SW480 cells in the miR‐338‐5p mimic group was markedly greater than that in the mimic NC group. Similarly, the motility, as represented by the migration ability, of SW620 cells after transfection with the miR‐338‐5p inhibitor was apparently inhibited (Figure 5K). Furthermore, the invasive capability of SW480 cells was apparently enhanced after upregulation of miR‐338‐5p but suppressed by knockdown of miR‐338‐5p (Figure 5L). These data suggest that miR‐338‐5p can promote cell behaviors related to CRC progression.

### 2.6 MiR‐338‐5p Reverses the Tumor‐Suppressive Effect of circFBXW4 on CRC Cells

**Figure 6:**
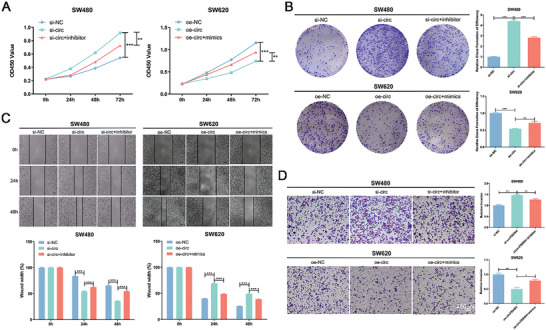
MiR‐338‐5p reversed the tumor‐suppressive effect of circFBXW4 on CRC cells. A) The results of CCK‐8 assays revealed that the effects on cell proliferation induced by circFBXW4 knockdown or overexpression were reversed by cotransfection with the miR‐338‐5p inhibitor or mimic, respectively. B) The results of colony formation assays revealed that the effects on cell proliferation induced by circFBXW4 knockdown or overexpression were reversed by cotransfection with the miR‐338‐5p inhibitor or mimic, respectively. C) The results of wound healing assays revealed that miR‐338‐5p inhibitor or miR‐338‐5p mimic transfection reversed the changes in the migration ability of CRC cells induced by circFBXW4 knockdown or overexpression, respectively. D) The results of Transwell assays revealed that miR‐338‐5p inhibitor or miR‐338‐5p mimic transfection reversed the changes in the invasion ability of CRC cells induced by circFBXW4 knockdown or overexpression, respectively. *P < 0.05, **P < 0.01, ***P < 0.001.

To further explore whether circFBXW4 suppresses the progression of CRC cells by sponging miR‐338‐5p, a series of rescue assays were performed by cotransfecting the miR‐338‐5p mimic or inhibitor with the circFBXW4‐OE plasmid or si‐circFBXW4. The results of the CCK‐8 and colony formation assays demonstrated that silencing circFBXW4 increased the viability of SW480 cells, while transfection of the miR‐338‐5p inhibitor partially attenuated the viability‐promoting effect of circFBXW4 silencing. Similarly, circFBXW4 overexpression decreased the viability of SW620 cells, and this inhibition was reversed by transfection of the miR‐338‐5p mimic (**Figure** **6**A,B). The wound healing assay results demonstrated that silencing circFBXW4 expression promoted wound healing in SW480 cells, while transfection of the miR‐338‐5p inhibitor partially attenuated this promoting effect of circFBXW4 downregulation. Similarly, overexpression of circFBXW4 inhibited wound healing in SW620 cells, and miR‐338‐5p mimic transfection partially attenuated this inhibitory effect (Figure 6C). The Transwell invasion assay results indicated that silencing circFBXW4 expression enhanced the invasion ability of SW480 cells and that transfection of the miR‐338‐5p inhibitor partially attenuated the increase in invasion induced by circFBXW4 downregulation. However, circFBXW4 overexpression decreased the invasion ability of SW620 cells, and this increase was partially offset by transfection of the miR‐338‐5p mimic (Figure 6D). Taken together, the above results suggest that circFBXW4 promotes the malignant progression of CRC cells partially by sponging miR‐338‐5p.

### 2.7 SLC5A7 is a Functional Target of miR‐338‐5p

**Figure 7:**
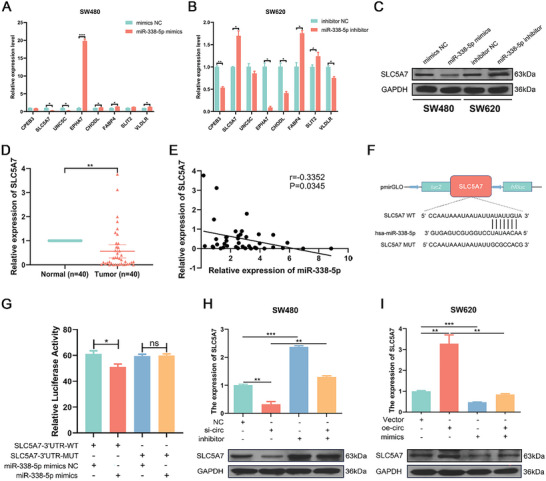
CircFBXW4 regulates SLC5A7 expression by acting as a sponge for miR‐338‐5p. A) Relative expression of candidate mRNAs in SW480 cells transfected with the miR‐338‐5p mimic. B) Relative expression of candidate mRNAs in SW620 cells transfected with the miR‐338‐5p inhibitor. C) Relative protein levels of SLC5A7 in CRC cells transfected with the miR‐338‐5p mimic or inhibitor. D) The relative expression of SLC5A7 in CRC tissues and matched adjacent normal tissues was determined by qRT‒PCR (n = 40). E) Pearson correlation analysis between the expression levels of miR‐338‐5p and SLC5A7 in our own patient cohort (n = 40). F) Schematic illustration of the SLC5A7‐WT and SLC5A7‐MUT luciferase reporter vectors. G) Relative luciferase activity was measured in 293T cells after cotransfection with SLC5A7‐WT or SLC5A7‐MUT and the miR‐338‐5p mimic or NC. H) The relative mRNA and protein expression levels of SLC5A7 were measured by qRT‒PCR and western blotting in SW480 cells transfected with si‐NC or si‐circFBXW4 with or without the miR‐338‐5p inhibitor. I) The relative mRNA and protein levels of SLC5A7 were measured by qRT‒PCR and western blotting in SW620 cells transfected with vector or the circFBXW4‐OE plasmid with or without the miR‐338‐5p mimic. The data are shown as the means ± SDs; *P < 0.05, **P < 0.01, ***P < 0.001.

To identify the potential downstream targets of miR‐338‐5p, we performed bioinformatics analyses using the TargetScan and miRDB databases. Then, we intersected these target genes with the differentially expressed mRNAs identified by our RNA‐seq analysis, and 8 overlapping genes (CPEB3, SLC5A7, UNC5C, EPHA7, CHODL, FABP4, SLIT2, and VLDLR) were obtained. qRT‒PCR analysis showed that transfection of the miR‐338‐5p mimic apparently downregulated SLC5A7 and UNC5C expression in SW480 cells (**Figure** **7**A). However, the expression levels of FABP4, SLC5A7 and SLIT2 were markedly increased by transfection of the miR‐338‐5p inhibitor (Figure 7B). Therefore, SLC5A7 was selected as the target of miR‐338‐5p for subsequent studies. The results of western blotting further indicated that miR‐338‐5p overexpression apparently suppressed SLC5A7 expression, while SLC5A7 protein expression was elevated after transfection with the miR‐338‐5p inhibitor (Figure 7C). We further measured the expression level of SLC5A7 in 40 paired CRC and adjacent normal tissues and found that SLC5A7 expression was frequently suppressed in CRC tissues compared with adjacent normal tissues (*P* < 0.01, Figure 7D). Pearson correlation analysis revealed that the miR‐338‐5p level was negatively correlated with the SLC5A7 level in CRC tissues (*r* = −0.335, *P* = 0.034, Figure 7E). To verify the binding between miR‐338‐5p and SLC5A7, we performed dual luciferase reporter assays. Luciferase reporter plasmids containing sequences with the wild‐type and mutant SLC5A7 binding sites (SLC5A7‐WT and SLC5A7‐MUT, respectively) were constructed (Figure 7F). Upregulation of miR‐338‐5p apparently suppressed luciferase activity in cells transfected with the wild‐type vector, while luciferase activity in cells transfected with the mutant vector did not change significantly (Figure 7G).

### 2.8 CircFBXW4 Regulates SLC5A7 Expression by Targeting miR‐338‐5p

To explore whether circFBXW4 plays its tumor‐suppressive role through the circFBXW4/miR‐338‐5p/SLC5A7 axis, miR‐338‐5p rescue experiments were performed. Si‐circFBXW4 and the miR‐338‐5p inhibitor were transfected separately or simultaneously into SW480 cells. The qRT‒PCR and western blot analyses indicated that circFBXW4 silencing apparently reduced the mRNA and protein levels of SLC5A7 and that knockdown of miR‐338‐5p markedly increased the mRNA and protein levels of SLC5A7. Cotransfection of si‐circFBXW4 and the miR‐338‐5p inhibitor reversed the downregulation of SLC5A7 expression caused by si‐circFBXW4 transfection (Figure 7H). Similarly, the circFBXW4‐OE plasmid and the miR‐338‐5p mimic were transfected into SW620 cells separately or simultaneously. CircFBXW4 overexpression apparently increased the mRNA and protein levels of SLC5A7, while transfection of the miR‐338‐5p mimic exerted the opposite effect. When the circFBXW4‐OE plasmid and the miR‐338‐5p mimic were cotransfected into SW620 cells, the increase in SLC5A7 expression caused by circFBXW4 overexpression was attenuated (Figure 7I). Overall, the results above suggest that circFBXW4 could regulate SLC5A7 expression by targeting miR‐338‐5p, thus suppressing CRC progression.

### 2.9 CircFBXW4 Suppresses the Growth of CRC In Vivo

**Figure 8:**
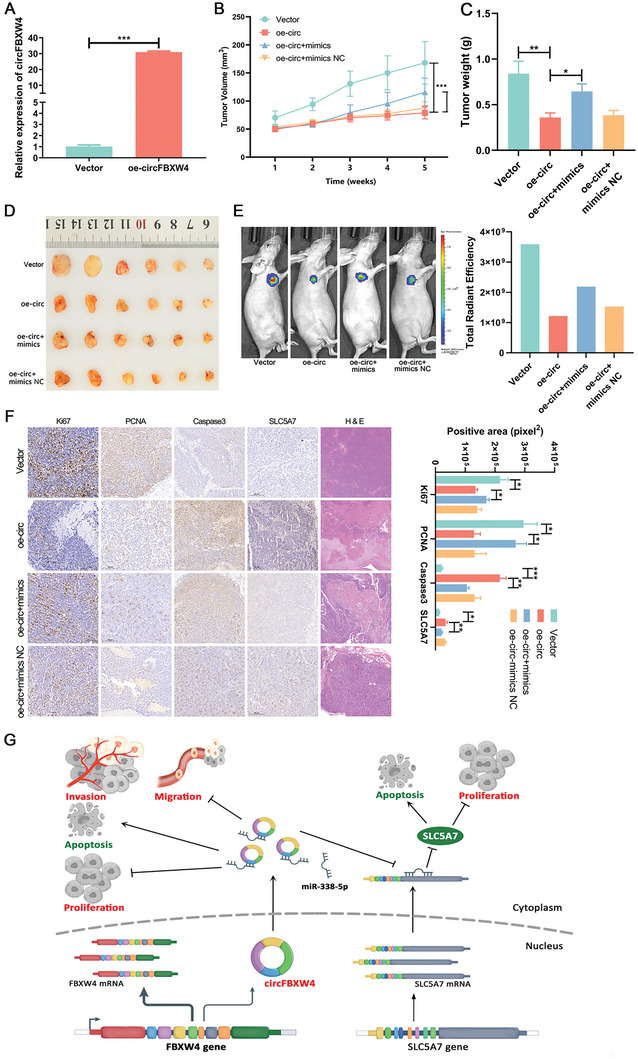
CircFBXW4 suppresses CRC tumor growth in vivo. A) The expression of circFBXW4 was measured by qRT‒PCR in SW620 cells stably overexpressing circFBXW4. B) Comparison of tumor volume curves in the circFBXW4‐OE, vector, circFBXW4‐OE + miR‐338‐5p mimic, circFBXW4‐OE + miR‐338‐5p control groups. C) Comparison of tumor weights in the circFBXW4‐OE, vector, circFBXW4‐OE + miR‐338‐5p mimic, circFBXW4‐OE + miR‐338‐5p control groups. D) Representative images of CRC xenograft tumors in each group. E) Bioluminescent Imaging (BLI) of mice in the circFBXW4‐OE, vector, circFBXW4‐OE + miR‐338‐5p mimic, circFBXW4‐OE + miR‐338‐5p control groups. F) Changes in Ki67, PCNA, Caspase3, and SLC5A7 expression in xenograft tumors were detected by IHC staining. G) Schematic diagram describing the proposed regulatory mechanism underlying the circFBXW4‐dependent regulatory network and the miR‐338‐5p/SLC5A7 axis in colorectal carcinogenesis. CircFBXW4 functions as a sponge for miR‐338‐5p and in turn promotes the expression of SLC5A7. *P < 0.05, **P < 0.01, ***P < 0.001.

To validate the tumor‐suppressing role of circFBXW4 by sponging miR‐338‐5p in vivo, we constructed SW620 cell lines with stable circFBXW4 overexpression via lentiviral transduction and verified the overexpression efficiency of circFBXW4 (**Figure** **8**A). SW620 cells infected with the circFBXW4‐OE lentivirus, vector control, circFBXW4‐OE lentivirus + miR‐338‐5p mimic, and circFBXW4‐OE lentivirus + miR‐338‐5p control were injected subcutaneously into the dorsal surface of BALB/c nude mice to evaluate the effects of circFBXW4 on tumor growth by sponging miR‐338‐5p in vivo. Every 7 days, the volumes of the subcutaneous tumors were calculated. CircFBXW4 overexpression in SW620 apparently decreased the tumor volume and weight (Figure 8B–D), which were partially reversed by miR‐338‐5p overexpression. Similarly, the xenograft tumors were assessed using the IVIS Spectrum Imaging System. The results showed that circFBXW4 overexpression effectively decreased the size of xenograft tumor, miR‐338‐5p overexpression partially abolished the inhibitory effect of circFBXW4 on tumor growth (Figure 8E). Furthermore, immunohistochemical (IHC) staining illustrated that decreased the expression of Ki67, PCNA, and increased the expression of Caspase3 and SLC5A7 were observed in the overexpressed circFBXW4 group as compared with the control group (Figure 8F). MiR‐338‐5p overexpression partially reversed the effect of circFBXW4 on the expression of proliferation‐ and apoptosis‐related proteins in tumor tissues of mice (Figure 8F). Collectively, these results suggest that circFBXW4 functions as a sponge for miR‐338‐5p and in turn promotes the apoptosis and inhibits the proliferation, invasion, and migration of CRC cells. The tumor‐suppressive effects may be exerted through the circFBXW4/miR‐338‐5p/SLC5A7 axis (Figure 8G).

## 3 Discussion

CircRNAs usually have a low abundance in cells due to the inefficiency of backsplicing. With the wide application of RNA‐seq technologies and bioinformatic techniques, numerous unique circRNAs have been discovered.^[^ ^28^ ^]^ Compared with microarray analysis, RNA‐seq analysis not only has the advantages of high throughput and data accuracy but also can reveal novel circRNAs, and it is currently the most technically effective and cost‐effective method for screening circRNAs. In this work, for the first time, we identified a novel circRNA termed circFBXW4, which is dramatically downregulated in CRC tissues and cell lines, by RNA‐seq and bioinformatics analyses. According to the circBase database and UCSC Genome Browser, circFBXW4 is derived from the FBXW4 gene on chromosome 10 and is composed of exons 2–5 (chr10: 103427642–103436193) via backsplicing. We characterized circFBXW4 as a novel circRNA by confirming its physical circular structure. Next, we further confirmed the backsplicing site of circFBXW4 by Sanger sequencing. These results indicated that circFBXW4 is a circRNA formed by backsplicing at the posttranscriptional level. CircRNAs are somewhat resistant to exonuclease digestion due to their lack of free ends, and they are generally more stable than linear RNAs. We confirmed the stability of circFBXW4 by an RNase R assay and inhibition of RNA synthesis by an actinomycin D assay, thus indirectly proving the ring structure of circFBXW4.

CircRNAs often exhibit tissue‐specific expression patterns.^[^ ^15^ ^]^ Some circRNAs are dysregulated in CRC and closely linked to clinical traits such as tumor size, histological type, tumor stage, and postoperative recurrence.^[^ ^29^, ^30^, ^31^ ^]^ In rapidly proliferating cancer cells, many circRNAs seem to be downregulated. This phenomenon may be due to the slow biogenesis of circRNAs, which are thus unable to reach homeostasis in rapidly proliferating cells, resulting in a dilution effect during cell division.^[^ ^32^ ^]^ However, circRNAs that have not reached steady‐state abundances in rapidly proliferating cells may be robust markers, because their expression levels reflect the aggressiveness of the cancer. In addition, circRNAs exist in various human bodily fluids at relatively high steady‐state levels.^[^ ^17^, ^33^ ^]^ These properties indicate the strong potential of circRNAs as biomarkers. Wang et al.^[^ ^34^ ^]^ demonstrated that circSPARC expression was elevated in both the plasma and tissues of patients with CRC and that high circSPARC expression was correlated with advanced tumor stage, lymph node metastasis, and worse prognosis. Similarly, circ3823 expression was apparently elevated in CRC tissues and serum and was linked to tumor grade, intestinal wall invasion depth, and lymph node metastasis.^[^ ^30^ ^]^ The ROC curve showed that the AUC was 0.807 (95% CI: 0.690‐0.924), indicating that circ3823 in serum may be a novel diagnostic biomarker for CRC. Further survival analysis showed that patients with elevated circ3823 expression had a worse prognosis.^[^ ^30^ ^]^ In this work, we found that circFBXW4 was apparently downregulated in CRC and that circFBXW4 downregulation was linked to poor tumor differentiation and advanced clinical stage. The AUC value of circFBXW4 was 0.879, which was much higher than that of SLC5A7 (AUC of 0.767), suggesting that circFBXW4 has potential value as a diagnostic biomarker for CRC. Interestingly, lower expression of circFBXW4 was observed in high‐grade glioma, and its potential diagnostic value was also demonstrated in glioma, with a high AUC value (AUC = 0.9467).^[^ ^25^ ^]^ Moreover, low circFBXW4 expression was associated with a short overall survival time. Based on the above results, circFBXW4 may be an important tumor suppressor underlying the initiation and progression of CRC and may have high value as diagnostic and prognostic marker for CRC.

Although circRNA research is a relatively young and controversial field, accumulating studies have placed circRNAs at the center of cancer biology by demonstrating their influence on several hallmarks of cancer.^[^ ^32^ ^]^ CircRNAs are involved in various characteristics of and processes in CRC cells, including cell viability, migration, invasion, cell cycle progression, apoptosis, ferroptosis, angiogenesis, and autophagy.^[^ ^30^, ^35^, ^36^, ^37^ ^]^ Guo et al.^[^ ^30^ ^]^ confirmed that circ3823 promoted the viability, angiogenesis, and metastasis of CRC cells via the miR‐30c‐5p/TCF7 axis. To further clarify the roles of circFBXW4 in the biological behaviors of CRC cells, we performed several functional experiments. CircFBXW4 overexpression suppressed the proliferation and metastasis and enhanced the apoptosis of CRC cells, while silencing circFBXW4 had the opposite effects. In addition, the results of our animal experiments verified the inhibitory effect of circFBXW4 on CRC tumor growth. Taken together, these findings indicate that circFBXW4 plays a vital role in the initiation and progression of CRC as a tumor suppressor.

Many studies have suggested that circRNAs play crucial regulatory roles in cancer initiation and progression. CircRNAs in the cytoplasm mainly act as miRNA sponges or interact with RBPs, while circRNAs in the nucleus mainly regulate transcription. Given that the majority of circRNAs contain miRNA response elements (MREs), they function mainly as miRNA sponges. Studies have shown that RNA molecules containing MREs can interact with and regulate each other through the competitive binding of miRNAs, thus serving as competing endogenous RNAs (ceRNAs).^[^ ^38^, ^39^ ^]^ CircRNAs can act as ceRNAs to bind miRNAs, thereby regulating the expression of the mRNAs targeted by these miRNAs at the posttranscriptional level. Additionally, the more MREs a circRNA contains, the more likely it is to function as a ceRNA.^[^ ^40^ ^]^ CircRNAs can bind to multiple sites of a specific miRNA, and a single circRNA can act on multiple miRNAs, thus exerting tumor‐suppressive or oncogenic effects.

In our attempt to elucidate the specific mechanism by which circFBXW4 affects the malignant behaviors of CRC cells, we confirmed that circFBXW4 was localized mainly in the cytoplasm through nuclear‐cytoplasmic separation and FISH experiments. Therefore, we investigated the mechanism by which circFBXW4 suppresses malignant behaviors of CRC cells from the perspective of miRNA sponging. First, we predicted the miRNAs that might bind to circFBXW4 by bioinformatic algorithms, and we then performed functional experiments. We found that miR‐338‐5p expression in CRC tissues was evidently upregulated and was negatively correlated with circFBXW4 expression. Furthermore, we discovered that circFBXW4 had a negative regulatory effect on miR‐338‐5p expression. Then, the direct binding of circFBXW4 and miR‐338‐5p was validated by dual luciferase reporter and RNA pull‐down assays. These data suggest that circFBXW4 can bind to miR‐338‐5p and negatively regulate its expression. Accumulating evidence suggests that miR‐338‐5p can function as a target of circRNAs.^[^ ^41^, ^42^, ^43^ ^]^ Shen et al.^[^ ^41^ ^]^ reported that hsa_circ_00 32683 could sponge miR‐338‐5p and reduce the inhibition of its target RTN4, thus suppressing the progression of hepatocellular carcinoma. Yao et al.^[^ ^42^ ^]^ found that circ_00 30167 could act as a sponge of miR‐338‐5p to upregulate the expression of its target WIF1, which attenuated the malignant progression of pancreatic cancer cells. In CRC, circRNAs also serve as miR‐338‐5p sponges. For example, Yang et al.^[^ ^43^ ^]^ showed that hsa_circ_013 7008 attenuates the malignant phenotype of CRC cells by sponging miR‐338‐5p.

To further explore the function of miR‐338‐5p in CRC, we performed several functional assays. Upregulation of miR‐338‐5p enhanced the proliferation and metastasis of colon cancer cells, while downregulation of miR‐338‐5p had the opposite effects. Numerous studies have shown that miR‐338‐5p is abnormally expressed in multiple cancers and can function as an oncogene or a tumor suppressor.^[^ ^44^, ^45^, ^46^ ^]^ Chu et al.^[^ ^27^ ^]^ found that miR‐338‐5p overexpression could promote the metastasis of CRC cells. Another study^[^ ^43^ ^]^ also showed that miR‐338‐5p expression was elevated in CRC tissues and could increase the viability and promote the metastasis of CRC cells, consistent with our results. After confirming that circFBXW4 can sponge miR‐338‐5p, we further revealed through a series of rescue experiments that circFBXW4 affects the changes in malignant behaviors of CRC cells via miR‐338‐5p. Transfection of the miR‐338‐5p mimic partially reversed the suppressive effects of elevated circFBXW4 expression on the proliferation and metastasis of CRC cells. Similarly, transfection of the miR‐338‐5p inhibitor partially attenuated the suppression of CRC cell viability and metastasis induced by circFBXW4 downregulation. Similar effects were also observed in xenograft models. Collectively, these results strongly support that circFBXW4 inhibits the malignant behaviors of CRC cells by sponging miR‐338‐5p.

As both circRNAs and miRNAs are ncRNAs, they must perform their biological functions by regulating their target proteins. Therefore, we screened for potential targets of miR‐338‐5p in public databases. The subsequent qPCR results showed that SLC5A7 was frequently suppressed in CRC tissues compared with adjacent normal tissues. Pearson correlation analysis revealed a negative correlation between the miR‐338‐5p and SLC5A7 levels in CRC tissues. Furthermore, qPCR and western blot analyses revealed that miR‐338‐5p negatively regulated SLC5A7 expression. Subsequently, we confirmed that miR‐338‐5p can directly bind to SLC5A7 by a dual luciferase reporter assay. SLC5A7, also called choline transporter 1 (CHT1), is a Na+/Cl–dependent choline cotransporter.^[^ ^47^ ^]^ SLC5A7 plays a vital role in normal neuromuscular junction signaling, and its mutations often lead to dominant motor neuropathies.^[^ ^48^, ^49^ ^]^ A recent study^[^ ^50^ ^]^ reported that SLC5A7 plays a crucial role in tumor suppression in CRC. This group first analyzed public databases and found that SLC5A7 was apparently downregulated in CRC tissues and that its expression was closely linked to TNM stage and prognosis. Moreover, IHC staining demonstrated that SLC5A7 was apparently downregulated or not expressed in CRC tissues. Survival analysis revealed that patients with elevated SLC5A7 expression had longer OS and disease‐free survival times. Overexpression of SLC5A7 suppressed the proliferation and enhanced the apoptosis of CRC cells. Mechanistically, SLC5A7 directly interacts with and modifies p53 to promote the expression of p53. SLC5A7 can also disrupt the interaction between p53 and MDM2 in wild‐type p53 CRC cells, promote p53 expression and inhibit ubiquitin‐mediated degradation of the p53 protein.

Based on the above research results, circFBXW4 can bind to and negatively regulate miR‐338‐5p, while miR‐338‐5p can bind to and negatively regulate SLC5A7. Therefore, we hypothesized that a circFBXW4/miR‐338‐5p/SLC5A7 regulatory axis exists in CRC. To verify this hypothesis, we designed a response experiment for circFBXW4 and miR‐338‐5p. Transfection with the miR‐338‐5p inhibitor reversed the downregulation of SLC5A7 expression caused by circFBXW4 knockdown. Similarly, upregulation of miR‐338‐5p reversed the overexpression of SLC5A7 caused by circFBXW4 overexpression. These results suggest that circFBXW4 regulates SLC5A7 expression by competitively binding to miR‐338‐5p.

In summary, we identified a novel circRNA, circFBXW4, through RNA‐seq and comprehensively explored its expression level, clinical significance, biological function, and regulatory mechanism in CRC. CircFBXW4 was dramatically downregulated in CRC tissues and cell lines, and its expression level was positively linked to tumor differentiation and negatively correlated with TNM stage and overall survival in CRC patients. Mechanistically, circFBXW4 competitively binds to miR‐338‐5p to abolish its inhibitory effect on SLC5A7, thereby suppressing CRC progression. This study elucidates the mechanism by which circFBXW4 inhibits the progression of CRC by the miR‐338‐5p/SLC5A7 axis and not only provides new insights into the pathogenesis of CRC but also identifies a potential therapeutic target for CRC.

## 4 Experimental Section

### CRC tissues

In total, 86 paired CRC tissues and adjacent noncancerous tissues were collected from the Department of Gastrointestinal Surgery, Renmin Hospital of Wuhan University, between 2019 and 2020 for RNA‐seq and qRT‒PCR analyses. Collection of the tissues was approved by the Ethics Committee of Renmin Hospital of Wuhan University, and written informed consent was provided by the patients. The CRC tissue was quickly appraised after isolation and placed in liquid nitrogen until use. The pathological diagnosis of CRC was independently confirmed by examination of the tissue samples from CRC patients by two pathologists. None of the patients received neoadjuvant therapy before surgery. Clinical data of CRC patients, including sex, age, tumor site, differentiation status, surgery type, histological type, tumor size, TNM stage, CEA level, and CA19‐9 level, were collected.

### RNA‐seq Analysis and Data Processing

It was randomly selected CRC and adjacent nontumor tissues from 5 of 86 patients with stage II or III CRC for RNA‐seq analysis. Total RNA from CRC tissues was extracted by Takara RNAiso Plus, and the extracted total RNA was sampled for quality inspection. Then, RNA‐seq libraries were constructed. In brief, prior to construction of RNA‐seq libraries, designed DNA probes were used to remove ribosomal RNA. Then, the RNA was fragmented for first‐strand cDNA synthesis, and the strand‐specific method was used to label the second strand of cDNA by adding dUTP during synthesis. After completion of end repair, poly(A) tails were added, the ligation product was purified, the fragments were sorted by size, and the libraries were amplified. Before PCR amplification, second‐strand cDNA was removed by uracil DNA glycosylase (UDG), and after amplification, the RNA‐seq libraries were purified by incubation with magnetic beads. Finally, whole‐transcriptome RNA‐seq, including circRNAs, long noncoding RNAs (lncRNAs), and mRNAs, was performed using the NovaSeq 6000 platform in PE150 mode.

After the original data were acquired, FastQC software was used to evaluate its quality, including the base quality and sequence quality. Cutadapt software was then used to filter out low‐quality sequences, and Hisat2 software was used to compare the retained reads to known sequences in a public database. For circRNA, DCC (https://github.com/dieterich‐lab/DCC%60) was used as the integrated appraisal software. Finally, the gene expression data were filtered and normalized to obtain the normalized gene expression profile for subsequent analysis.

### Cell lines and Cell Culture

The human normal colon epithelial cell line NCM460 and colon cancer cell lines (SW480, SW620, HT29, and Caco2) were obtained from the Cell Bank of the Chinese Academy of Sciences (Shanghai, China). SW480 and SW620 cells were cultured using L‐15 medium, while HT29 cells were cultured using McCoy's 5A medium containing 10% fetal bovine serum (FBS). Caco2 cells were cultured in MEM containing 20% FBS, and all cells were cultured in medium supplemented with 1% penicillin‒streptomycin at 37 °C with 5% CO_2_.

### RNA/Genomic DNA (gDNA) Extraction and qRT‒PCR

Total RNA from CRC cells or tissue samples was extracted with TRIzol reagent (Invitrogen, CA, USA), and gDNA from CRC tissues was extracted with a MiniBEST Universal Genomic DNA Extraction Kit (Takara, Japan). Total RNA was reverse transcribed into cDNA with miRNA‐specific stem‒loop or random primers. Divergent and convergent primers specific for the circRNAs and linear mRNAs, respectively, were designed. All primer sequences were listed in Table S1 (Supporting Information). Real‐time PCR was performed with TB Green Premix Ex Taq II (Takara, Japan).

### Agarose Gel Electrophoresis

The PCR products were subjected to 2% agarose gel electrophoresis with 1×TAE buffer. An appropriate volume of each PCR product was mixed with 6× loading buffer mixture in each well, and 5 µL of DNA Marker I was added as a reference. After 30 to 40 min of protein separation by electrophoresis at 100 V, the gel was placed in the gel imaging system, and the DNA bands on the gel were observed.

### Treatment with RNase R and Actinomycin D

Total RNA from SW480 and SW620 cells was divided into 5 equal samples. One RNA sample without RNase R (Epicenter Technologies, USA) was used as the control group, and the other four RNA samples were incubated with 3 U µg^−1^ RNase R at 37 °C for various time intervals and then at 80 °C for 10 min to inactivate the RNase R. The expression levels of circFBXW4 and the corresponding linear FBXW4 mRNA were measured by qRT‒PCR. To prevent nascent RNA synthesis, cells were exposed to 2 µg mL^−1^ actinomycin D (Sigma, USA). Cell precipitates were collected after different time intervals, and RNA was extracted for reverse transcription and subsequent qRT‒PCR.

### Cell Transfection

To construct the circFBXW4 overexpression (circFBXW4‐OE) plasmid, circFBXW4 cDNA was inserted into the pCD2.1‐ciR vector. The circFBXW4 siRNA (si‐circFBXW4), miR‐338‐5p mimic, and miR‐338‐5p inhibitor sequences were designed and synthesized by GeneCreate (Wuhan, China). Transfection was performed using Lipofectamine 2000 (Invitrogen, USA). The si‐circFBXW4 sequences were listed in Table S2 (Supporting Information).

For stable overexpression of circFBXW4, the circFBXW4 sequence and a cyclization‐promoting sequence were inserted into the ZSGreen/Puromycin lentiviral vector. SW620 cells were spread in 6‐well plates at 50%–60% confluence. Culture medium and virus were added to the 6‐well plates, and the medium was replaced with fresh medium for 8–12 h. After infection for ≈72 h, the transduction efficiency was evaluated by fluorescence assessment. Then, the cells were inoculated into culture bottles. After the cells were fully adherent to the culture bottle wall, puromycin was added to screen for stably transduced cells. When all the cells in the normal group were nonviable, cell precipitates were collected, and the upregulation of circFBXW4 was validated by qRT‒PCR.

### Cell Proliferation Assays

For the CCK‐8 assay, 1 × 10^3^ CRC cells/well were seeded into 96‐well plates. After culture for different time periods, CCK‐8 solution (Dojindo, Japan) was added to each well, and the plates were incubated in a 5% CO_2_ incubator for 1–2 h. The absorbance was measured at a wavelength of 450 nm. For the colony formation assay, 200 cells/well were seeded into 6‐well plates and cultured for 2 weeks. Colonies were stained with crystal violet prior to image acquisition.

### Wound Healing Assay

CRC cells were seeded in 6‐well plates and cultured in a 5% CO_2_ incubator at 37 °C. When the cells were more than 90% confluent, scratches were manually generated with a 10 µL sterile pipette tip. Then, the 6‐well plate was placed under an inverted microscope for observation and photography, and the cells were placed in a 37 °C 5% CO_2_ incubator for further culture. The scratches were observed with an inverted microscope at 24 and 48 h, and the cell migration distance was calculated using ImageJ software.

### Transwell Migration and Invasion Assays

For the migration and invasion assays, the membranes in the upper chambers were uncoated or coated with Matrigel (BD Biosciences, USA), respectively. Then, 500 µL of L‐15 medium supplemented with 20% FBS was added to the lower chambers, and the upper chambers were placed in the 24‐well plate. CRC cells were resuspended in serum‐free medium. Then, 10 000 cells were added to the upper chambers and cultured at 37 °C in an incubator with 5% CO_2_. After incubation for 24 to 48 h, the cells were fixed and stained with 0.1% crystal violet. The migrated and invaded cells were counted under a microscope (Olympus Corporation).

### Cell Apoptosis Assays

CRC cells were stained with the Annexin V‐FITC/PI Apoptosis Detection Kit (BD Biosciences, Franklin Lakes, NJ, USA). The cells were digested with EDTA‐free trypsin and washed with precooled PBS. Next, they were stained with Annexin V‐FITC and propidium iodide (PI) for 15 min. Stained cells were analyzed using an Attune NXT Acoustic Focusing Flow Cytometer (Thermo Fisher Scientific, MA, USA).

### Mouse Xenograft Models

All animal experiments were approved by the Animal Management Committee of Renmin Hospital of Wuhan University. A total of 24 BALB/c nude mice (6‐8 weeks old) were randomly assigned to the circFBXW4‐OE, vector control, circFBXW4‐OE + miR‐338‐5p mimic, or circFBXW4‐OE + miR‐338‐5p control groups (n = 6 per group). SW620 cells (2 × 10^6^) were subcutaneously injected into the right dorsal surface of each mouse. After tumor cell inoculation, the volumes of the tumors were calculated every 7 days by the following equation: volume = (length × width^2^)/2. When the nude mice developed cachexia or the maximum tumor diameter was ≈1.5 cm, the mice were sacrificed. The tumors were excised for gross observation, weighed, photographed, and analyzed by IHC staining.

### Immunohistochemistry

Mouse xenograft tissues were fixed with 4% paraformaldehyde for 24 h and embedded in paraffin. The embedded tissues were sliced into 3 µm thick sections and then processed for hematoxylin and eosin (H&E) staining and IHC staining. The primary antibodies used to detect Ki‐67, PCNA, and Caspase3 were purchased from ABclonal (China), and the primary antibody used to detect SLC5A7 was purchased from ProteinTech (China). The sections were examined under an Olympus inverted microscope (Japan). The immunoreactivity in each section was assessed by two experienced pathologists. The degree of positivity was determined according to the staining intensity and the distribution of stained cells.

### FISH

The FISH probe for circFBXW4 was synthesized by GenePharma (Shanghai, China). In brief, CRC cells were seeded on a cell slide, fixed, and permeabilized in 0.5% Triton X‐100. Then, the slide was blocked in prehybridization solution for 30 min, and the probe mixture was then added for hybridization overnight in an incubator at 37 °C. After hybridization, nuclei were stained with DAPI, and a laser confocal microscope was used for observation and photography.

### Dual Luciferase Reporter Assay

Sequences containing the predicted binding sites of miR‐338‐5p with circFBXW4 and SLC5A7 were inserted into a dual luciferase reporter vector, which was cotransfected with the miR‐338‐5p mimic into 293T cells. After 48 h of incubation, the cells were subjected to luciferase activity measurement with a Dual‐Luciferase Reporter Assay System.

### RNA Pull‑Down Assay with Biotinylated circFBXW4

The biotin‐labelled circFBXW4 probe and the oligo probe were synthesized by GeneCreate (Wuhan, China). In brief, the biotin‐labelled circFBXW4 probe was added to streptavidin magnetic beads and incubated for 15–30 min to produce probe‐coated magnetic beads. Next, cell lysates were thoroughly mixed with the magnetic bead complexes and incubated at 4 °C for 30–60 min. After elution of the magnetic bead‐RNA‒RNA complexes, total RNA was extracted, and reverse transcription and qRT‒PCR were then performed. The sequence of the circFBXW4 probe was listed in Table S3 (Supporting Information).

### Western Blotting

Equal amounts of total protein in samples were separated by SDS‒PAGE and electroblotted onto a PVDF membrane. After blocking, the membrane was incubated first with a primary antibody targeting SLC5A7 (1: 1000, ABclonal, China, A8247) and subsequently with a secondary antibody. Immunoreactions were detected with a chemiluminescence imaging system (Bio‐Rad, USA) with ECL reagent (Millipore, Germany).

### Statistical Analysis

SPSS 26.0, R (version 4.2.0), and GraphPad Prism 8 software were used for data processing. Continuous variables are presented as means ± standard deviations (SDs), and differences between two groups were compared with two‐tailed Student's *t* test. Differences between categorical variables were analyzed with the chi‐square test. Pearson correlation coefficients were calculated to examine correlations. ROC analysis was applied to evaluate the diagnostic value of circFBXW4 for CRC screening. For survival analysis, the expression level of circFBXW4 was treated as a binary variable, and the patients were divided into groups with high and low circFBXW4 expression. Kaplan‒Meier analysis was performed using SPSS. Statistical significance was accepted for values of *P* < 0.05.

## Conflict of Interest

The authors declare no conflicts of interest.

## Author Contributions

W.S., J.F., and J.W. contributed equally to this work. W.S. and T.F. conceived and designed the study. W.S., J.F., J.W., J.R., R.X., and C.K. collected clinical tissue samples and clinicopathological data. W.S. executed the majority of experiments. W.S. and C.K. interpreted the data and performed the statistical analysis. W.S. wrote the manuscript. W.S. and T.F. revised the manuscript. All authors reviewed the manuscript.

## Supporting information

Supporting Information

## Data Availability

The data that support the findings of this study are available from the corresponding author upon reasonable request.
