# Supplementary material for: CircFBXW4 Suppresses Colorectal Cancer Progression by Regulating the MiR‐338‐5p/SLC5A7 Axis
Source: Adv Sci (Weinh). 2024 Mar 10;11(18):2300129. doi: 10.1002/advs.202300129 (PMC11095154; doi:10.1002/advs.202300129)
Supplement: Supplementary file 1 — Supporting Information [file ADVS-11-2300129-s001.pdf]

ADVANCED SCIENCE
Open Access

Supporting Information

for *Adv. Sci.*, DOI 10.1002/advs.202300129

CircFBXW4 Suppresses Colorectal Cancer Progression by Regulating the MiR-338-5p/SLC5A7 Axis

*Wei Song, Jincheng Fu, Jing Wu, Jun Ren, Rensheng Xiang, Can Kong and Tao Fu**

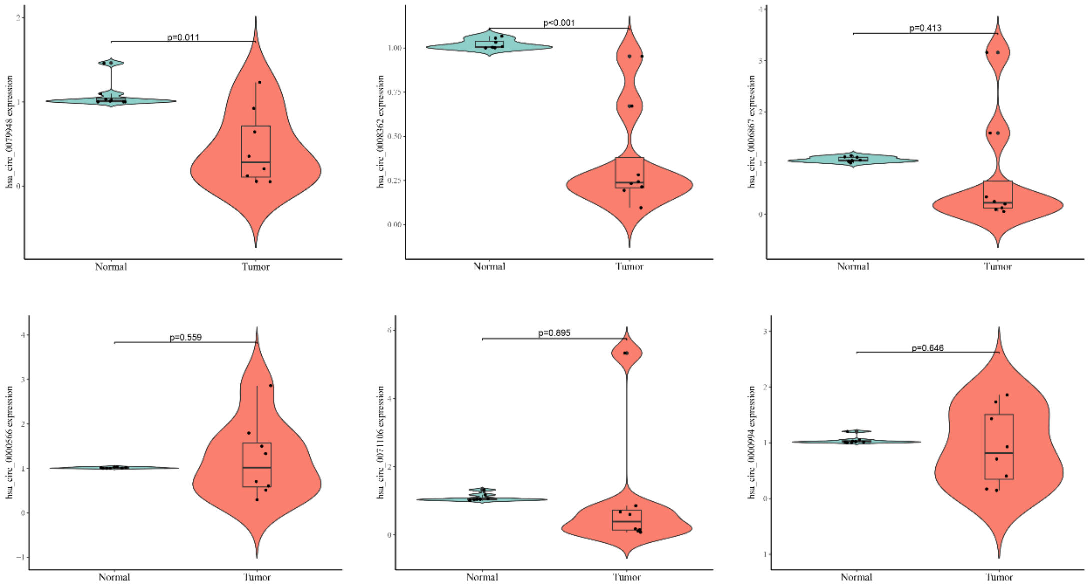

Figure S1 The expression levels of 6 circRNAs were measured by qRT–PCR in tissues from 8 paired CRC tissues and adjacent normal tissues.

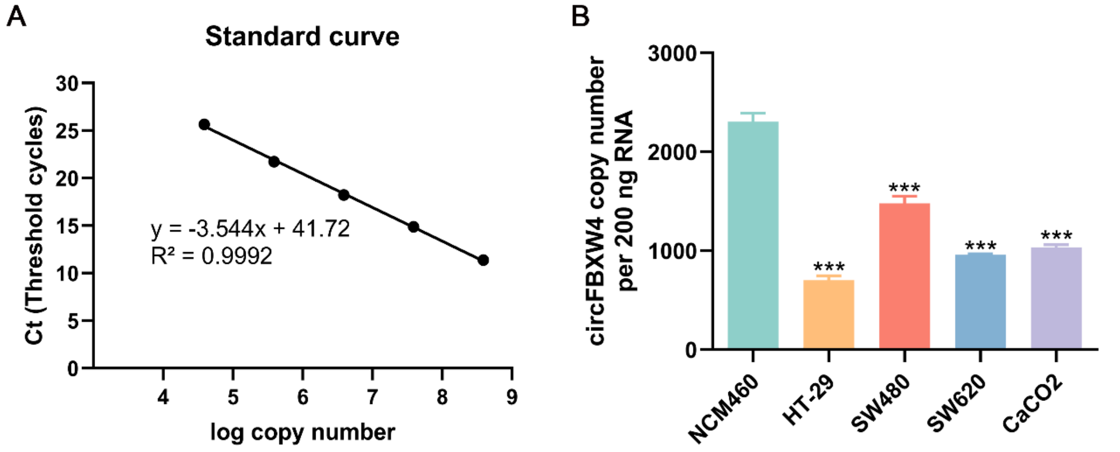

Figure S2 The absolute expression of circFBXW4 in CRC cell lines. A. Standard curve of circFBXW4 in absolute quantification. B. Copy number of circFBXW4 in CRC cell lines. Data were showed as mean ± SD. $^{**}P < 0.01$, $^{***}P < 0.001$.

Table S1. Primer sequences used for qRT–PCR analysis.

Gene	Primer Sequences (5’-3’)
hsa_circ_0000566	F: TGCAAGGACTAAAAGCTATAGGAAG<br>R: ACACGAGGCATTTTCACTTTGTT
hsa_circ_0071106	F: GGGACGGAGAGGTGTTCTTT<br>R: TTTCTTCCGATCAGCAGCTT

hsa_circ_0008362	F: AGCCCATTACTCAGGATGACC<br>R: GACTGCATCTCCACTTCAGCA
hsa_circ_0000994	F: TCGAGGAAATGTTATCGTTCCA<br>R: TCCAACTGTCACAACCTAACAAT
hsa_circ_0006867	F: CACGATAGCAGGACGGAGTTA<br>R: TGCAGCCCTTCTGTATATGCC
hsa_circ_0001380	F: TTCCGGCCACCCATTGATTT<br>R: GGCCGTCGTCTTTTAGGAGC
hsa_circ_0079948	F: GGGACAAAGATGAAAGCAAACA<br>R: GAAGGGAGACTCGGAAGCAG
hsa_circ_0007609	F: GACGAGAGGGAATAGCCTAACAT<br>R: ATGTCATCTGAGGTTGCGTTCT
FBXW4	F: AGTGAAGGAACGAGTGAAG<br>R: GTAGGCCAGGATGAAATTA
hsa-miR-324-3p	RT: CTCAACTGGTGTCGTGGAGTCGGCAATTCA<br>GTTGAGCCAGCAGC<br>F: ACACTCCAGCTGGGCCCACTGCCCCAGGTGC
hsa-miR-338-5p	RT: CTCAACTGGTGTCGTGGAGTCGGCAATTCA<br>GTTGAGCACTCAGC<br>F: ACACTCCAGCTGGGAACAATATCCTGGTGC
hsa-miR-1913	RT: CTCAACTGGTGTCGTGGAGTCGGCAATTCA<br>GTTGAGTGGCAGCA<br>F: ACACTCCAGCTGGGTCTGCCCCCTCCGCTG
hsa-miR-3187-3p	RT: CTCAACTGGTGTCGTGGAGTCGGCAATTC<br>AGTTGAGCCGCGCAG<br>F: ACACTCCAGCTGGGTTGGCCATGGGGCT
hsa-miR-4656	RT: CTCAACTGGTGTCGTGGAGTCGGCAATTC<br>AGTTGAGACAGGCCT<br>F: ACACTCCAGCTGGGTGGGCTGAGGGCAGGAG
hsa-miR-4793-3p	RT: CTCAACTGGTGTCGTGGAGTCGGCAATTC

	AGTTGAGAGCCAGCC<br>F: ACACTCCAGCTGGGTCTGCACTGTGAGTTGG
U6	F: CTCGCTTCGGCAGCACA<br>R: AACGCTTCACGAATTGTGCGT
CPEB3	F: GATGCCTGCCTAGAAGAAGATGG<br>R: GCACTGGCTTGTCCTTGATGG
SLC5A7	F: GTGGAACCTTGCCACCTAAA<br>R: AGCTGAGGGTCATGCTCTGT
UNC5C	F: TGTTGCTCTCTATGTTGGGATTGTG<br>R: TCTTGTCTTGCTGCCTTGATGTTC
EPHA7	F: AAGCAGGCTACCAGCAAAAA<br>R: GGTCAGATGGAGCCCTGTAA
CHODL	F: ATCACCAACCAACTGCCAATCC<br>R: TGTTACACCTGTCATCATTCCACTG
FABP4	F: AGGAAAGTCAAGAGCACCATAACC<br>R: CACCACCAGTTTATCATCCTCTCG
SLIT2	F: TCCCCACAAATCTTCCAGAG<br>R: AGCGTAGTCCTTGGAAAGCA
VLDLR	F: TGGTCGCTGTATTACGCTGTTG<br>R: ATATGGCACTGTTCTGGGCTTTC
GAPDH	F: GGAGTCCACTGGCGTCTTCA<br>R: GTCATGAGTCCTTCCACGATACC

Table S2. Si-circFBXW4 sequences used in this study.

**siRNA**		**Primer Sequence (5'-3')**
si-circFBXW4-1	sense	GCCCAUUACUCAGGAUGACCA
si-circFBXW4-1	antisense	UGGUCAUCCUGAGUAAUGGGC
si-circFBXW4-2	sense	GAGGAUGAUUCUCUGUACAUA
si-circFBXW4-2	antisense	UAUGUACAGAGAAUCAUCCUC

Table S3. Probe sequences of circFBXW4 used in this study.

siRNA	Primer Sequence (5'-3')
Biotin-circFBXW4 probe	CACTGGGACACTGGTCATCCTGAGTAATGG GCTGATAGC
NC-Biotin	AAACAGTACTGGTGTGTAGTACGAGCTGAA GCTAC
